# Comparison of capecitabine and oxaliplatin with S-1 as adjuvant chemotherapy in stage III gastric cancer after D2 gastrectomy

**DOI:** 10.1371/journal.pone.0186362

**Published:** 2017-10-17

**Authors:** Jang Ho Cho, Jae Yun Lim, Jae Yong Cho

**Affiliations:** Division of Medical Oncology, Department of Internal Medicine, Gangnam Severance Hospital, Yonsei University College of Medicine, Seoul, Korea; National Cancer Center, JAPAN

## Abstract

**Aim:**

To compare capecitabine and oxaliplatin (XELOX) with S-1 as adjuvant chemotherapy in stage III gastric cancer after D2 gastrectomy.

**Methods:**

Clinical data from 206 patients who received XELOX or S-1 regimens as adjuvant chemotherapy in stage III gastric cancer were collected. Patients were divided into 2 groups according to regimen; the groups were XELOX (n = 114) and S-1 monotherapy (n = 92).

**Results:**

3-year disease-free survival (DFS) was higher in the S-1 group than in the XELOX group (66.6% vs 59.1%; p = 0.636). 3-year overall survival (OS) was 75.6% in the S-1 group and 69.6% in the XELOX group (p = 0.495). But, the difference was not statistically significant. Especially, for patients with stage IIIC disease, 3-year overall survival was 55.2% in the XELOX group and 39.0% in the S-1 group (hazard ratio, HR 0.50, 95% confidence interval, CI 0.23–1.10; p = 0.075). In multivariate analysis, N stage (HR, 5.639; 95% CI, 1.297–24.522; p = 0.021) and cycle completion as planned (HR, 5.734; 95% CI, 3.007–10.936; p<0.001) were independent predictors of overall survival.

**Conclusion:**

Adjuvant XELOX and S-1 regimen did not prove anything superior for stage III gastric cancer in this study. But, XELOX had a tendency to be superior to S-1 in stage IIIC gastric cancer after D2 gastrectomy although the difference was not statistically significant. N stage and cycle completion as planned were prognostic factors.

## Introduction

Gastric cancer is the third most common cause of cancer-related mortality worldwide, with 951,000 new cases and 723,000 deaths per year [1]. In Asian countries, gastric cancer has a high incidence and is the most prevalent malignancy in Korea [2]. The mainstay of treatment for operable gastric cancer is surgery. However, recurrence rates are still high [3, 4]. Even after curative resection, in stage II and III disease, a considerable proportion of patients experienced recurrence. In Japan and South Korea, gastrectomy with extended lymphadenectomy (D2 gastrectomy) has been the standard surgical treatment for many years [5, 6]. Based on the 15-year follow-up results of a large Dutch D1D2 trial, which showed a reduction in gastric cancer-specific deaths with extended surgery [7], D2 gastrectomy is now recommended in Europe [8] and the USA [9] for resectable gastric cancer.

Adjuvant chemotherapy is the standard treatment for resectable gastric cancer and reduces the number of cancer-specific deaths [10, 11]. Although various regimens for adjuvant chemotherapy have been implemented to prevent postoperative recurrence, no regimens have been clearly recommended for adjuvant chemotherapy after D2 gastrectomy, which has been established as the standard procedure for advanced gastric cancer [12]. Furthermore, the preferred therapy differs by geographical region. In the UK and other European countries, perioperative chemotherapy is recommended [8]. In the USA, the recommended adjuvant therapy is chemoradiotherapy [9]. These recommendations are based on the UK Medical Research Council Adjuvant Gastric Infusional Chemotherapy (MAGIC) trials [13] and US Intergroup-0116 [14]. However, both studies assessed the survival benefits of adjuvant therapy after only limited dissection of the regional lymph nodes. The surgical method for gastric cancer can influence the results of postoperative chemotherapy [15].

Until now, there have been two important adjuvant chemotherapy trials for gastric cancer, which showed a survival benefit with adjuvant chemotherapy after D2 gastrectomy compared with surgery alone. One is the Adjuvant Chemotherapy Trial of S-1 for Gastric Cancer (ACTS-GC) study [11, 12] and the other is the Capecitabine and Oxaliplatin Adjuvant Study in Stomach Cancer (CLASSIC) study [16]. The ACTS-GC study in Japan, which collected data on patients with stage II and III gastric cancer who had received D2 gastrectomy, showed that S-1 chemotherapy leads to an 11% increase in the 5-year overall survival (OS) rate compared with patients who had undergone surgery alone (72% vs. 61%, HR, 0.67; 95% CI, 0.540–0.828) [12]. The CLASSIC study in South Korea, which collected data on patients with stage II, IIIA, and IIIB gastric cancer who had also received D2 gastrectomy, showed a 3-year disease-free survival (DFS) benefit with capecitabine plus oxaliplatin (XELOX) adjuvant chemotherapy compared with surgery alone (74% vs. 60%, HR, 0.56; 95% CI, 0.44–0.72; p<0.001) [16].

There is no standard regimen for adjuvant chemotherapy. Although many studies have been conducted to determine the optimal adjuvant chemotherapy regimen, it is still controversial. In the CLASSIC study, XELOX showed a greater benefit in patients with N1 or N2 nodal status than in those whose disease was limited to N0. Therefore, XELOX is probably more effective than S-1 in patients with aggressive disease status. However, S-1 is recognized as standard adjuvant chemotherapy for patients with resected gastric cancer in Japan, and is widely used in Asian countries because of its convenient administration method and good efficacy [11]. We aimed to compare capecitabine and oxaliplatin (XELOX) with S-1 as adjuvant chemotherapy in stage III gastric cancer after D2 gastrectomy.

## Materials and methods

### Patients

We collected the data of 3,928 patients who underwent surgery for gastric cancer between January 2011 and December 2013 at the Yonsei University Medical Center in South Korea (Severance Hospital, n = 3,337; Gangnam Severance Hospital, n = 591). Among these patients, eligible patients were ambulatory, aged 18 years or older, had histologically confirmed disease, had American Joint Committee on Cancer[17] stage III disease, had undergone D2 or more extensive lymph-node dissection with R0 resection, had no hepatic, peritoneal, or distant metastasis, and no tumor cells in peritoneal fluid on cytologic analysis. Patients were excluded only if they had received chemotherapy, immunotherapy, or radiotherapy for gastric cancer.

Of the 3,928 patients who underwent surgery for gastric cancer, there were 206 who met the eligibility criteria and received XELOX or S-1. Patient information was obtained from outpatient clinical or admission records and information regarding patient survival was obtained from the Korean National Statistics Registry Database. The protocols were approved by the Yonsei University Health System Institutional Review Board.

Patients who were treated with XELOX received 8 cycles of oral capecitabine (1000 mg/m^2^ twice daily on days 1–14 of each cycle) plus intravenous oxaliplatin (130 mg/m^2^ on day 1 of each cycle) every 3 weeks. Patients who were treated with S-1 received a daily dose of 80, 100, or 120 mg in 2 separate doses. The dose of S-1 was determined based on body surface area. S-1 was administered for 4 weeks, followed by 2 weeks of rest. This 6-week cycle was repeated during the first year after surgery. If patients had serious hematologic or nonhematologic toxic effects, dose reductions or interruptions were allowed. In cases of oxaliplatin-related neurological adverse events, capecitabine monotherapy was allowed. However, oxaliplatin monotherapy was not permitted. Relative dose intensity was the ratio of the delivered dose intensity to the planned dose intensity and was expressed as a percentage. A relative dose intensity of 100% indicates that the drug was administered at the planned dose, without delay and without cancellations.

Tumor assessment was conducted using abdominal computed tomography (CT) or magnetic resonance imaging (MRI) every 2 or 3 cycles of treatment. If symptoms or signs of recurrence or a new gastric cancer appeared, we immediately performed imaging studies, including ultrasonography, CT, gastrointestinal radiography series, and endoscopy. Adverse events were graded according to the National Cancer Institute’s Common Terminology Criteria for Adverse Events (version 3.0). DFS was defined as the time from the date of curative surgery for gastric cancer to the date of recurrence of the original gastric cancer, the development of a new gastric cancer, or death from any cause. OS was defined as the time from the date of curative surgery for gastric cancer to the date of death from any cause or the last follow-up.

### Statistical analyses

All statistical analyses were conducted using IBM SPSS ver.21.0 (IBM Co., Armonk, NY). A chi-square test was used to compare the demographics between treatment arms for discrete variables. For continuous variables, a two-tailed Student’s t test was used. The Kaplan-Meier method was used to estimate the cumulative survival. Estimates of treatment effect were calculated as hazard ratios (HRs) with 95% confidence intervals (CIs). Study treatment groups were compared with a two-sided log-rank test. A P value of less than 0.05 was considered to indicate statistical significance. Significant variables in the univariate analysis were entered into multivariate analysis using the Cox proportional hazards model.

## Results

### Clinical characteristics

**Fig 1:**
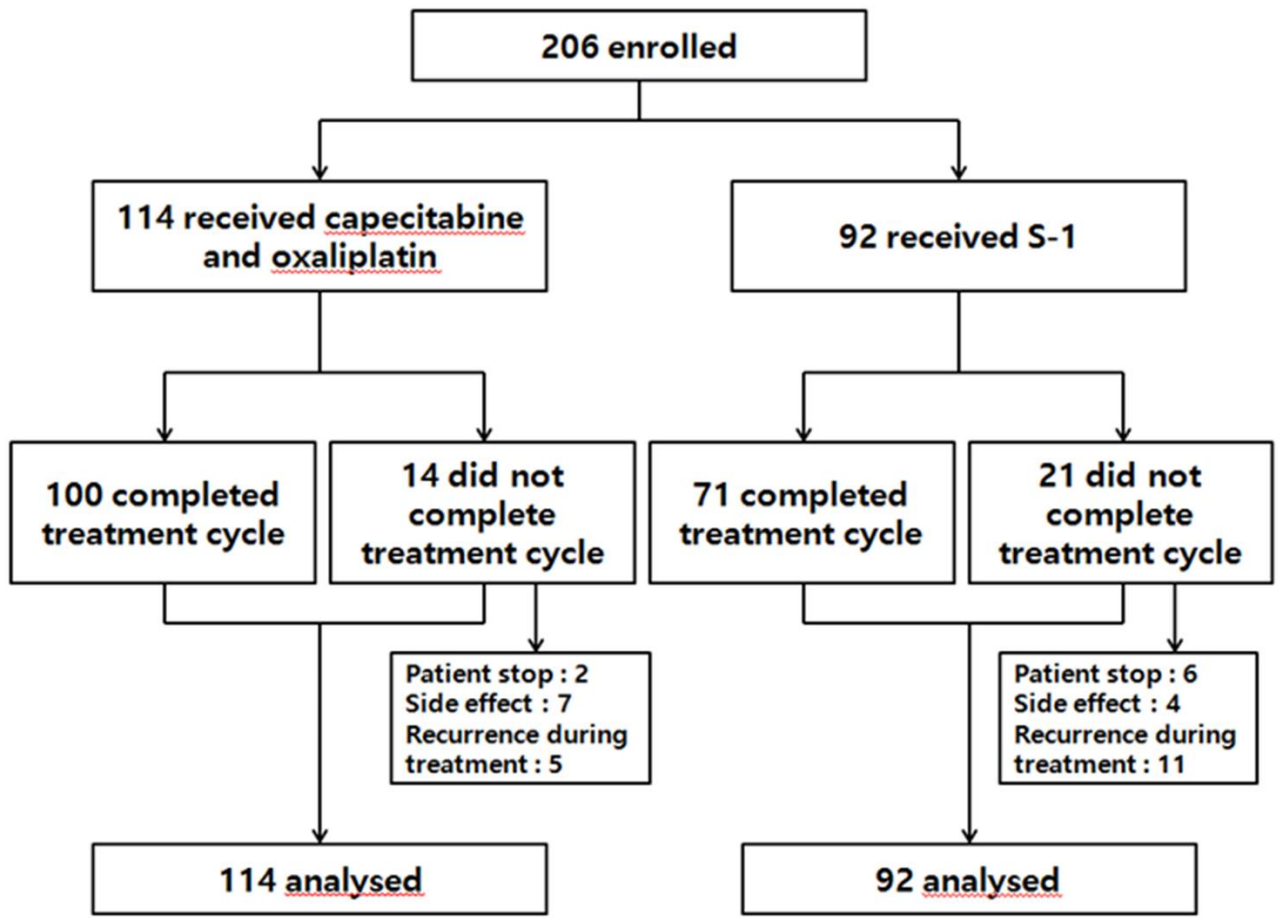
Study profile.

The median follow-up duration for all patients was 21.0 months until the OS data cutoff date (Jan 31, 2015), by which time all patients except for one had discontinued treatment. Fig 1 shows the study profile. A total of 87.7% (100/114) of the XELOX patients and 77.2% (71/92) of the S-1 patients completed the treatment. Fourteen patients receiving XELOX did not complete treatment. The reasons for this were that two patients stopped treatment at their own discretion, seven patients experienced severe adverse events, and five patients relapsed during XELOX treatment. Twenty one patients receiving S-1 did not complete treatment. The reason was that six patients stopped treatment at their own discretion, four patients experienced severe adverse events, eleven patients relapsed during S-1 treatment.

The clinical characteristics of patients are listed in Table 1. Comparison of the group of patients who received XELOX revealed that they were significantly younger than those in the S-1 group (median age, 56.29 vs. 68.69 years; p<0.001). Patients in the XELOX group also had higher incidence of perineural invasion, (86.8% vs. 72.8%, p<0.001). Except for this, patient demographic and baseline disease characteristics were similar in each group. Also, there was no difference in survival when these two factors were considered.

**Table 1:** Baseline patient characteristics.

Characteristic	XELOX (n = 114)	TS-1 (n = 92)	p-value
**Sex**			0.703
**Male**	76 (66.7%)	59 (64.1%)	
**Female**	38 (33.3%)	33 (35.9%)	
**Age (years)**	56.29±11.06	68.69±11.01	<0.001
**<60**	65 (57.0%)	15 (16.3%)	
**60–69**	35 (30.7%)	25 (27.2%)	
**70–79**	14 (12.3%)	41 (44.6%)	
**80–89**	0 (0.0%)	11 (12.0%)	
**ECOG PS**^#^			0.360
**0**	40 (35.1%)	38 (41.3%)	
**1**	74 (64.9%)	54 (58.7%)	
**Family gastric cancer history**			0.449
**Yes**	20 (17.5%)	20 (21.7%)	
**No**	94 (82.5%)	72 (78.3%)	
Tumor location*			0.375
**Proximal**	16 (14.0%)	3 (3.3%)	
**Body**	58 (50.9%)	54 (58.7%)	
**Antrum**	31 (27.2%)	33 (35.9%)	
**Multiple or diffuse**	9 (7.9%)	2 (2.2%)	
**Bormann type (unknown: 2)**			0.912
**I**	6 (5.3%)	4 (4.3%)	
**II**	29 (25.4%)	21 (22.8%)	
**III**	53 (46.5%)	51 (55.4%)	
**IV**	25 (21.9%)	15 (16.3%)	
**Differentiation**			0.842
**Well or moderately-differentiated**	25 (21.9%)	25 (27.2%)	
**Poorly-differentiated or signet-ring cell**	86 (75.4%)	60 (65.2%)	
**Others**	3 (2.6%)	7 (7.6%)	
**Lauren type (unknown: 4)**			0.138
**Intestinal**	34 (29.8%)	41 (44.6%)	
**Diffuse**	66 (57.9%)	40 (43.5%)	
**Mixed**	12 (10.5%)	9 (9.8%)	
**Maximum tumor size (cm)**	6.50±3.76	5.76±2.43	0.090
**T stage**			0.360
**T1**	0 (0.0%)	0 (0.0%)	
**T2**	3 (2.6%)	4 (4.3%)	
**T3**	20 (17.5%)	19 (20.7%)	
**T4**	91 (79.8%)	69 (75.0%)	
**N stage**			0.074
**N0**	0 (0.0%)	0 (0.0%)	
**N1**	15 (13.2%)	21 (22.8%)	
**N2**	35 (30.7%)	28 (30.4%)	
**N3**	64 (56.1%)	43 (46.7%)	
**Lymphovascular invasion**			0.216
**YES**	88 (77.2%)	64 (69.6%)	
**NO**	26 (22.8%)	28 (30.4%)	
**Perineural invasion**			0.011
**YES**	99 (86.8%)	67 (72.8%)	
**NO**	15 (13.2%)	25 (27.2%)	

Data are mean (SD) or n (%).

^#^ECOG PS, Eastern Cooperative Oncology Group Performance Status;

*Antrum is the lower third, body is the middle third, and proximal is the upper third.

### Treatment outcome

**Fig 2:**
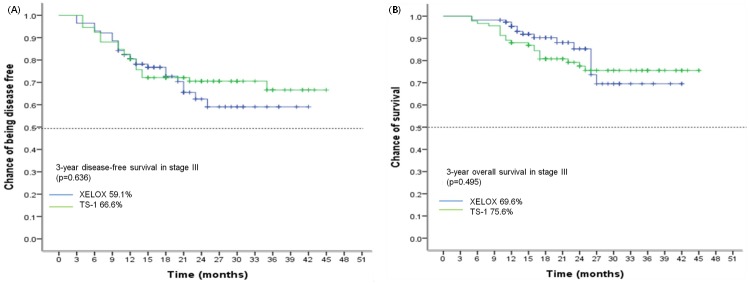
3 year survival for stage III patients in XELOX and S-1 group. (A) DFS. (B) OS.

At the cutoff date, 32 patients (28.1%) in the XELOX group and 27 patients (29.3%) in the S-1 group had relapsed. 3-year DFS was higher in the S-1 group than in the XELOX group (66.6% vs 59.1%; p = 0.636; Fig 2A). Fifteen patients (13.2%) in the XELOX group died and 20 patients (21.7%) in the S-1 group died. 3-year OS was 69.6% in the XELOX group and 75.6% in the S-1 group (p = 0.495; Fig 2B). But, the difference was not statistically significant.

**Fig 3:**
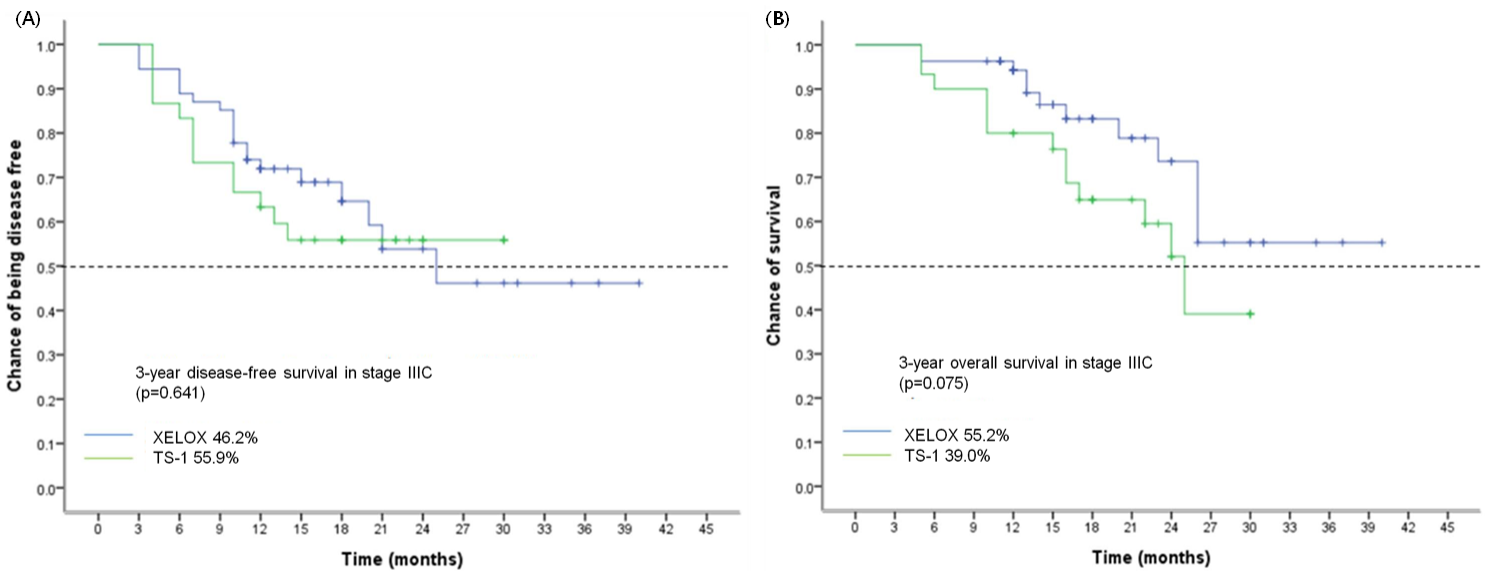
3 year survival for stage IIIC patients in XELOX and S-1 group. (A) DFS. (B) OS.

**Fig 4:**
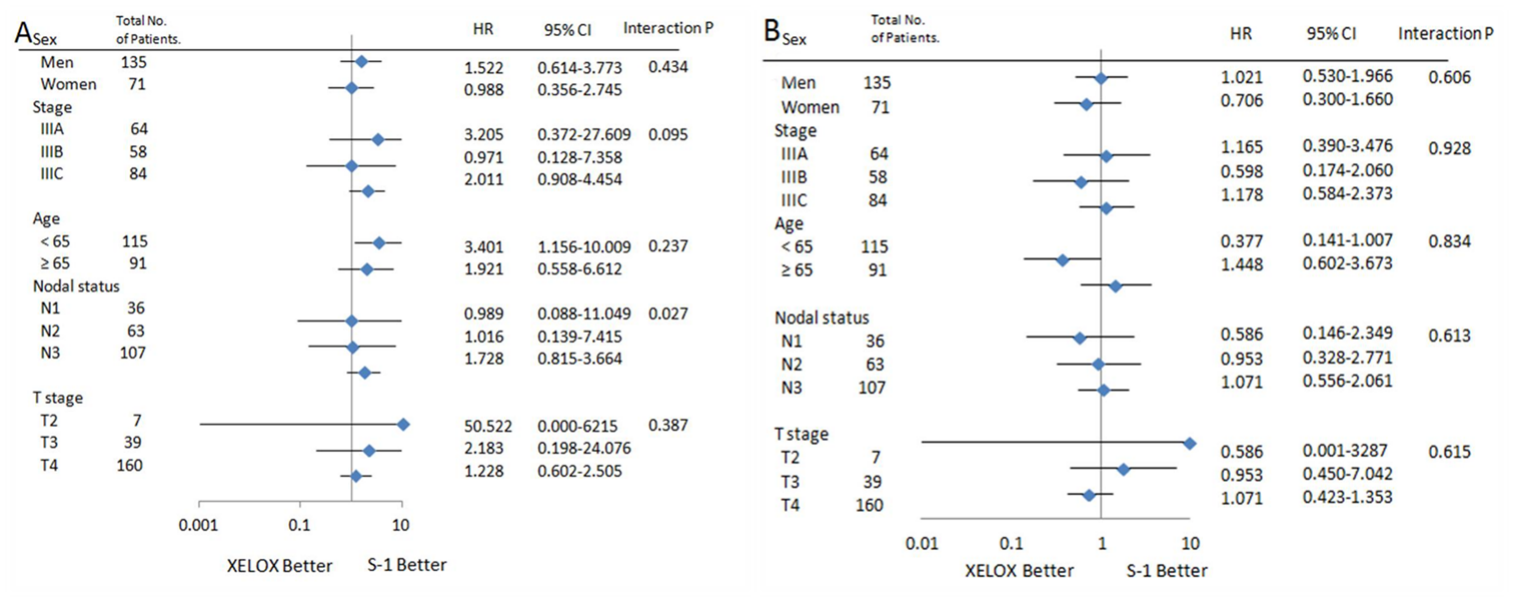
The forest plot graphs for subgroup analysis for eligible population. (A) OS. (B) DFS.

Fig 2 shows 3-year DFS and OS for patients with stage III disease. None of the two treatment groups proved superiority. Subgroup analysis showed 3-year DFS (Fig 3A) and 3-year OS (Fig 3B) for XELOX compared with S-1 in patients with stage IIIC disease. 3-year OS was 55.2% in the XELOX group and 39.0% in the S-1 group (HR 0.50, 95% CI 0.23–1.10; p = 0.075). As shown in Table 2, overall survival between the two treatment groups varied according to the nodal status (interaction p = 0.027). Subgroup analysis of OS and DFS in eligible patients showed in Table 2, Fig 4A and 4B. But, there was no meaningful result in subgroup analysis.

**Table 2:** Subgroup analysis: OS and DFS in study patients.

	Total no. of Patients	Overall survival			Disease-free survival		
		HR	95% CI	Interaction P	HR	95% CI	Interaction P
**Sex**							
**Men**	135	1.522	0.614–3.773	0.434	1.021	0.530–1.966	0.606
**Women**	71	0.988	0.356–2.745		0.706	0.300–1.660	
**Stage**							
**IIIA**	64	3.205	0.372–27.61	0.095	1.165	0.390–3.476	0.928
**IIIB**	58	0.971	0.128–7.358		0.598	0.174–2.060	
**IIIC**	84	2.011	0.908–4.454		1.178	0.584–2.373	
**Age**							
**< 65**	115	3.401	1.156–10.01	0.237	0.377	0.141–1.007	0.834
**≥65**	91	1.921	0.558–6.612		1.488	0.602–3.673	
**Nodal status**							
**N1**	36	0.989	0.088–11.05	0.027	0.586	0.146–2.349	0.613
**N2**	63	1.016	0.139–7.415		0.953	0.328–2.771	
**N3**	107	1.728	0.815–3.664		1.071	0.556–2.061	
**T stage**							
**T2**	7	50.52	0.000–6215	0.387	50.52	0.000–6215	0.615
**T3**	39	2.183	0.198–24.08		1.780	0.450–7.042	
**T4**	160	1.228	0.602–2.505		0.756	0.423–1.353	

The sites of gastric cancer recurrence were the peritoneum, hematogenous sites, locoregional sites, and lymph nodes (Table 3). Rates of relapse were lower in the S-1 group than in the XELOX group for the peritoneum and lymph nodes; however, this was not significant.

**Table 3:** Site of first relapse*.

Site	S-1 (n = 92)		XELOX (n = 114)		HR	95% CI	p-value
	No.	%	No.	%			
**Total no. of relapses**	27	29.3	32	28.1	-	-	
**Peritoneum**	10	10.9	18	15.8	0.643	0.322–1.284	0.211
**Lymph nodes**	2	2.2	4	3.5	0.815	0.330–2.013	0.657
**Hematogenous**	11	12.0	8	7.0	1.300	0.688–2.454	0.419
**Local**	4	4.3	2	1.8	1.089	0.179–6.629	0.926

*Some patients had a first relapse at more than one site.

Upon univariate analysis of all patients, N stage and chemotherapy cycle completion as planned were prognostic factors associated with survival. After adjusting for covariates in multivariate analysis, N stage (HR, 0.205; 95% CI, 0.089–0.473; p = <0.001), and cycle completion as planned (HR, 5.575; 95% CI, 2.801–11.096; p<0.001) were independent predictors of OS (Table 4).

**Table 4:** Univariate and multivariate analysis showing factors associated with overall survival in 206 patients.

Variable	Univariate		Multivariate	
	HR (95% CI)	p-value	HR (95% CI)	p-value
Sex	1.324 (0.677–2.590)	0.412		
Age	0.985 (0.958–1.013)	0.286		
ECOG	1.101 (0.541–2.241)	0.792		
Signet–ring cells	1.339 (0.406–4.419)	0.631		
Tumor size	1.088 (0.992–1.192)	0.073		
T stage	1.297 (0.175–9.597)	0.314		
N stage (N1,N2 vs N3)	0.201 (0.087–0.463)	<0.001	0.205 (0.089–0.473)	<0.001
Lymphovascular invasion	0.661 (0.298–1.463)	0.307		
Perineural invasion	0.566 (0.218–1.474)	0.244		
CEA	0.455 (0.183–1.128)	0.089		
CA 19–9	0.700 (0.338–1.448)	0.336		
**Cycle completed as planned**	5.583 (2.840–10.976)	<0.001	5.575 (2.801–11.096)	<0.001

HR, hazard ratio; CI, confidence interval; CEA, carcinoembryonic antigen; CA, cancer antigen.

Table 5 shows adverse events reported by 5% of patients or more. Almost two times as many grade 3 or 4 adverse events were reported in the XELOX group compared with the S-1 group (54/114 (47%) vs. 19/92 (21%)). In the XELOX group, the most commonly reported adverse events of any grade were neutropenia, thrombocytopenia, anemia, nausea, peripheral neuropathy, and diarrhea. In the S-1 group, the most commonly reported adverse events of any grade were neutropenia, anemia, thrombocytopenia, anorexia, diarrhea, nausea, skin hyperpigmentation and oral mucositis. The most common grade 3 or 4 adverse events in the XELOX group were neutropenia and nausea, while the most common in the S-1 group was neutropenia.

**Table 5:** Adverse events reported by ≥5% of patients.

	S-1 (n = 92)		XELOX (n = 114)	
	All grades	Grade 3 or 4	All grades	Grade 3 or 4
**Neutropenia**	66 (72%)	12 (13%)	84 (74%)	40 (35%)
**Thrombocytopenia**	21 (23%)	0 (0%)	73 (64%)	8 (7%)
**Anemia**	58 (63%)	0 (0%)	77 (68%)	1 (1%)
**Nausea**	13 (14%)	1 (1%)	32 (28%)	20 (18%)
**Peripheral neuropathy**	0 (0%)	0 (0%)	25 (22%)	0 (0%)
**Diarrhea**	16 (17%)	2 (2%)	17 (15%)	0 (0%)
**Hand-foot syndrome**	4 (4%)	0 (0%)	10 (9%)	1 (1%)
**Vomiting**	2 (2%)	0 (0%)	8 (7%)	6 (5%)
**Anorexia**	19 (21%)	2 (2%)	5 (4%)	3 (3%)
**Mucositis oral**	11 (12%)	0 (0%)	4 (4%)	0 (0%)
**Skin hyperpigmentation**	13 (14%)	0 (0%)	1 (1%)	0 (0%)

Data are n (%). Grades of adverse events were defined according to the Common Toxicity Criteria of the National Cancer Institute (version 4.0).

Because of adverse events, 75.4% (86/114) of patients in the XELOX group received dose reductions. In this group, 72.8% of patients (83/114) received oxaliplatin dose reductions, and 63.1% (74/114) had capecitabine dose reductions. Five patients stopped oxaliplatin because of severe adverse events, and they received capecitabine monotherapy. In the S-1 group, 30.4% of patients (28/92) received dose reductions. The median relative dose intensity was 71.8% for oxaliplatin, 77.2% for capecitabine, and 88.8% for S-1.

## Discussion

We showed that 1) Adjuvant XELOX was not superior to S-1 in stage III gastric cancer in terms of survival though there was a statistically non-significant trend to better survival in stage IIIC gastric cancer. 2) N stage and cycle completion as planned were prognostic factors. 3) Dose intensity with XELOX was lower than S-1 due to toxicities.

Subgroup analysis produced more meaningful results in this study. In particular, we focused on stage IIIC results. Subgroup analysis suggested that adjuvant XELOX was beneficial for stage IIIC. The data showed an improvement in OS with XELOX compared with S-1. Although the results were not statistically significant, it seems obvious that XELOX had superior OS compared with S-1 for patients with stage IIIC disease.

Across all patients, the S-1 regimen was associated with better 3-year DFS and OS than the XELOX regimen. But, the difference was not statistically significant. This study was retrospectively analyzed, so it is speculated that there is a selection bias between the two treatment groups.

This study used 3-year DFS and 3-year OS parameters because most recurrences happen within 3 years of surgery in gastric cancer [18]. The GASTRIC group meta-analysis of 17 trials also showed the clinical relevance of DFS for gastric cancer. The meta-analysis revealed an 18% relative risk reduction for both DFS and OS with adjuvant chemotherapy compared with surgery only [10].

In the present study, the adverse events in each chemotherapy regimen were analyzed. Grade 3 or 4 adverse events in the XELOX group were two times more frequent than in the S-1 group. Because of adverse events, the most common being neutropenia and nausea, 75.4% of patients who received the XELOX regimen and 30.4% of patients who received the S-1 regimen needed dose modification. The frequency, severity, and type of adverse events documented in our study were similar to those of the CLASSIC [16] and ACTS-GC [11] study. However, there were the patients who were treated with the XELOX regimen in our study experienced more hematologic adverse events (e.g. neutropenia, thrombocytopenia, and anemia) than those in the CLASSIC trial.

In conclusion, findings from our study support that XELOX had a tendency to be superior to S-1 in stage IIIC gastric cancer after D2 gastrectomy although the difference was not statistically significant and that XELOX regimen has manageable toxicity. Therefore, the XELOX regimen could be considered first in patients who have a high risk of recurrence, especially those with stage IIIC disease. Future prospective randomized trials are needed to investigate these results.
